# Hoarding Vaccines or Hedging Vaccine R&D Risks? — Motivation for Overbooking COVID-19 Vaccines in High-Income Countries

**DOI:** 10.34172/ijhpm.2024.8350

**Published:** 2024-02-27

**Authors:** Qi Shao

**Affiliations:** Department of Situation and Policy, Huaibei Normal University, Huaibei, China.

## Dear Editor,

 In the early stages of the COVID-19 pandemic, global supplies of vaccines were extremely tight, but some high-income countries (HICs) pre-ordered more vaccines than their nationals needed. We consider an entity overbook COVID-19 vaccines when the percentage of the national population that can be vaccinated with the pre-order vaccine reaches or exceeds 200% since most vaccines require only two doses,^1^ and not everyone is suitable for vaccination. There are five such entities as of January 29, 2021 (Figure). The overbooking of vaccines in HICs has led to complaints from other countries, who accused HICs of hoarding vaccines, which was an act of selfishness, shamelessness, and greed.^2-6^ However, people seem to overlook that the COVID-19 vaccine is unsuitable for hoarding because the COVID-19 vaccine’s shelf life is just 3-6 months,^7^ and the domestic demand is limited. So, why did HICs pre-order more vaccines than their population needed?

**Figure:**
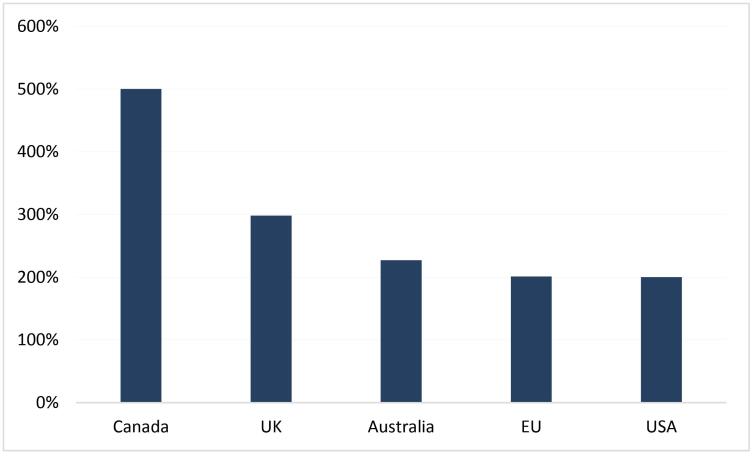
Percentage of National Population Vaccinated With Pre-ordered COVID-19 Vaccines in Entities. Source: Duke Global Health Innovation Center. COVID-19 VACCINE DATA. https://launchandscalefaster.org/node/64. Data as of January 29, 2021. These figures may be underestimated because some trading information is not publicised. Calculation formula: Total pre-order COVID-19 vaccine doses/Total population *100% = The percentage of the national population that can be vaccinated with the pre-order COVID-19 vaccine.

 If we understand the vaccine development process, we should know that vaccine development risks are very high. Vaccines, like most biologics, involve very large, complex molecules comprising component parts whose interactions are often unpredictable. Likewise, the human immune system is often unexpected in its responses to various stimuli and difficult to model.^8^ So, only about 7% of vaccine candidates can complete preclinical development, and only about 15% to 20% of candidates can go through clinical trials and receive market approval.^9^ Considering the feature of vaccine development, governments had to invest in a diverse portfolio to maximise the chances of finding a successful and effective vaccine as quickly as possible.^10^ For example, as of August 2020, the US government had invested up to $9 billion in 19 COVID-19 vaccine candidates spread among seven companies.^11^ The total number of vaccines pre-ordered by the governments generally has a certain redundancy to ensure enough vaccines are available even if some vaccine development fails, which some people misunderstand as hoarding vaccines. If some countries are lucky enough to receive more vaccines than their populations need, there is little use for the excess vaccines other than to donate or resell to vaccine-deficient countries.^11^ It is also true that those countries that donate the most vaccines are just the HICs that receive the most criticism during the pandemic. As of September 7, 2021, the United States, the United Kingdom, France, Canada, and Spain had donated 114.4 million, 6.9 million, 4.4 million, 1.4 million, and 1.3 million doses of COVID-19 vaccine to other countries bilaterally and through COVAX, respectively, placing them as the world’s most generous donors in the 1st, 5th, 6th, 7th, 8th places.^12^

 However, not all of the COVID-19 vaccines overbooked in HICs were donated or resold to vaccine-deficient countries, and many vaccines were discarded because they had expired. It is estimated that between March and August 2021, at least 15.1 million doses of the COVID-19 vaccine were discarded in the United States. By then, the vaccine was unavailable in many parts of the global South, with vaccination rates on the African continent below 10%.^13^ Even the surplus vaccine from HICs was eventually trans-shipped to vaccine-deficient countries, and they often had a shelf life of only a few weeks by the time they reached their destination because of the long trans-shipment cycle, so many expire before they could be used. This approach was almost tantamount to treating vaccine-deficient countries as repositories for soon-to-expire leftovers.^14^ Thus, while the primary motivation for HICs to overbook the COVID-19 vaccine was to hedge against the risk of vaccine development failure rather than hoarding the vaccine, this behaviour was still one of the major causes of the uneven distribution of vaccines globally. “No one is safe until everyone is safe.”^15^ All countries should unite to fight the pandemic. To optimise global vaccine allocation, countries with the capacity to develop the vaccine, once they have succeeded, should transfer patents and technology as soon as possible to countries with the capacity to produce vaccines, which can promote the localisation of vaccine production, improve production efficiency, reduce transaction costs and avoid research and development risks. International institutions should also work to lower barriers to the trade of vaccines and their intellectual property rights and to direct HICs to donate vaccines to fragile countries.

## Ethical issues

 Not applicable.

## Competing interests

 Author declare that he has no competing interests.

## Disclaimers

 I solemnly promise I submit the paper to be completed by me independently, never plagiarize, citing the results of other people’s data has been indicated sources. If this paper involves intellectual property disputes, I am fully responsible.

## Funding

 This work was supported by a school-level quality engineering project at Huaibei Normal University [grant number 2023jxyj044].
